# Unveiling the neuromechanical mechanisms underlying the synergistic interactions in human sensorimotor system

**DOI:** 10.1038/s41598-020-80420-z

**Published:** 2021-01-08

**Authors:** S. Honarvar, C. Kim, Y. Diaz-Mercado, K. Koh, H. J. Kwon, T. Kiemel, M. Caminita, J. O. Hahn, J. K. Shim

**Affiliations:** 1 Department of Mechanical Engineering, University of Maryland, College Park, MD 20742 USA; 2 School of Mechanical Engineering, Chonnam National University, Gwangju, 61186 South Korea; 3 School of Medicine, University of Maryland, Baltimore, MD 21201 USA; 4 Department of Kinesiology, University of Maryland, College Park, MD 20742 USA; 5 Program in Neuroscience and Cognitive Science, University of Maryland, College Park, MD 20742 USA; 6 Department of Mechanical Engineering, Kyung Hee University, Yongin-Si, Gyeonggi-do South Korea

**Keywords:** Motor control, Systems biology

## Abstract

Motor synergies are neural organizations of a set of redundant motor effectors that interact with one another to compensate for each other’s error and ensure the stabilization of a performance variable. Recent studies have demonstrated that central nervous system synergistically coordinates its numerous motor effectors through Bayesian multi-sensory integration. Deficiency in sensory synergy weakens the synergistic interaction between the motor effectors. Here, we scrutinize the neuromechanical mechanism underlying this phenomenon through spectral analysis and modeling. We validate our model-generated results using experimental data reported in the literature collected from participants performing a finger force production task with and without tactile feedback (manipulated through injection of anesthetic in fingers). Spectral analysis reveals that the error compensation feature of synergies occurs only at low frequencies. Modeling suggests that the neurophysiological structures involving short-latency back-coupling loops similar to the well-known Renshaw cells explain the deterioration of synergy due to sensory deprivation.

## Introduction

Humans have a sophisticated body structure with typically more degrees of freedom (DOF), i.e., motor effectors, than are required for successful task performance^1^. Therefore, theoretically, each motor task can be performed in an endless number of ways. One of the main issues in motor control is how the central nervous system (CNS) manages to select a solution from many apparently similar alternatives for performing a motor task^1^. There have been many attempts to answer the DOF problem. Among all these attempts, a well-known optimal feedback control theory^2^ suggests that CNS sets up feedback controllers that continuously convert sensory input to motor output based on the principle of minimum intervention, so that the feedback controllers correct only those deviations that intervene in the task^1–3^. The feedback nature suggests that the CNS can handle perturbations perceived by sensory systems. In other words, CNS does not look for a single optimal solution; it preferably utilizes a family of solutions known as synergies. The term “synergy” originates from Greek and means working together. In the context of neuroscience, synergies refer to the task-specific neural organization of a set of redundant motor effectors that work together to ensure stability and flexibility in task performance^4^. Such task-specific organizations have the features of load sharing and error compensation^4^. Load sharing refers to the distribution of the task over a set of motor effectors^5^. Error compensation refers to the fact that if one effector produces more or less than its expected share, other effectors change their contributions in a way that the task is performed correctly or at least better than if the effectors act independently^4^.

The creation of synergies is thought to be the mechanism by which the CNS controls its redundant motor effectors. CNS integrates a continuous stream of sensory information from multiple modalities and synergistically coordinates the motor effectors by sending appropriate motor commands to the effectors. The sensory and motor systems intimately interplay as a closed-loop feedback control system. The sensory feedback gathered from various modalities is often noisy and delayed, which may challenge the CNS to control our movements. Sometimes a movement duration is shorter than the sensory delay, and can cause the control system to correct for outdated and non-existent errors and lead the system to instability^3^. So, the way by which the CNS integrates noisy delayed sensory information and uses it to synergistically control its redundant motor elements for successful task performance has not become clear yet to the scientific community. Empirical studies have provided convincing evidence that humans optimally combine information from multiple sensory modalities in a way that enhances perception, which may be best described by Bayesian integration^6,7^. It has been shown that humans integrate visual and haptic information in a statistically optimal manner, similar to maximum-likelihood integration. More specifically, visual and haptic information tend to be weighted by the inverse of their variances, which reflect the reliability of each sensory modality. The more reliable modality contributes more to the information fed back to the CNS in order to enhance perception. It has been reported that premotor, parietal, and subcortical areas of the brain contain multimodal neurons. These neurons responded stronger^8^ and faster^9^ by the presence of multi-sensory stimuli than that of uni-sensory stimuli. It also has been found that reaction time in response to visual and tactile stimuli are faster than those to unimodal stimuli^10^.

Recent studies show that the cutaneous afferent feedback plays a critical role in providing information on the current state of the system and enabling the CNS to control the hand for gripping and grasping^11–18^. These results have shown that grip forces were consistently excessive with the hand subject to digital anesthesia during sub-maximal force production tasks. Besides, it has been demonstrated that cutaneous feedback enhances the synergistic interaction between fingers in an isometric multi-finger force production task^17^.

Although experimental descriptions of human movement have revealed a variety of possible mechanisms responsible for neural control of movement, it is still challenging to comprehend how the complete system might work. Understanding the neural control mechanism of human behavior through a dynamic modeling approach can contribute to the elucidation of the systemic mechanisms observed in experimental studies and allow for testing hypotheses that cannot be directly validated in experiments.

Several studies have been conducted to develop neural mechanism models of sensorimotor interactions and synergistic control scheme^19^ by incorporating modern control techniques such as feedforward control^20^, pure feedback control, optimal feedback control^2^, adaptive control^21^, and neural networks^22,23^. However, none of these have established a relationship between sensory integration and the motor synergy associated with motor redundancy. Our previous simulation of the central back-coupling hypothesis (CBC) demonstrated that a simple feedforward neurophysiological structure without sensory feedback could theoretically represent synergistic interaction between effectors^24^. This neurophysiological structure, which is similar to the well-known Renshaw cell^25^, involves short-latency feedback and tunable back-coupling gains that ensure error compensation between motor effectors. However, this kind of simulation cannot explain how sensory degradation can affect motor function and possibly the synergistic interaction between effectors. Previous studies have shown that abnormalities in the sensory information due to pathophysiological conditions can lead to deterioration of motor function as well as synergetic interaction between motor effectors^11–14^.

The aim of this study is to shed light on the neuromechanical mechanism underlying motor synergy and the degradation of motor synergy due to sensory deprivation through spectral analysis and modeling. The presented model represents the neuromechanical control of CNS in a multi-digit finger force production task and the synergistic interaction between fingers under two conditions: with and without tactile feedback. This task is one of the representative synergetic movements in the human body, where the combined force of all fingers is controlled by incorporating the redundant DOF of multiple digits.

In this model, we represent the sensorimotor system as a closed-loop feedback control system where tactile and visual feedbacks are integrated using Bayesian rule and fed back to CNS. In our work, the CNS model includes elements of the CBC^24^, including sharing, self and lateral inhibition and enslaving—the fact that activation of one finger invokes co-activation of others due to the mechanical interconnection between fingers^22^. Including the elements of CBC in the CNS model allows for a synergistic interaction between fingers according to the motor synergy theory and the CBC hypothesis. A proportional-derivative-lag (PD-lag) compensator is used to replicate the control aspect of the CNS. Finally, we represent the dynamics of individual fingers using a second-order linear system. We validate the results of model-generated finger force production against human-generated finger force production using a hierarchical variability decomposition (HVD) technique^17^. Human-generated finger force production was previously collected^17^ from participants performing the task of finger force production with and without tactile feedback (manipulated through digital anesthesia). Here, we construct our model based on previous neurophysiological knowledge and motor synergy theory. Thus, our intention is to reproduce the general trends in the experimental data rather than the minute details.

In our endeavor to investigate the human sensorimotor system's underlying control mechanism, we scrutinize different components of the model and address the following questions: (1) How does the deprivation of one sensory modality affects sensory delays and latencies? (2) Which aspects of CNS change due to deprivation of one sensory modality? (3) Which aspect of the sensorimotor system is responsible for the reduced synergy after sensory deprivation?

To construct the model and answer the questions mentioned above, we extract the important features of finger force experimental data, including the finger force trajectories, as well as the distributions for finger force variances, finger force sharing ratios, enslaving, and some control design characteristics. Our results reveal that synergies' error compensation feature occurs through the counter-directional drifts in finger force trajectories, which allows the total force to remain constant. Indeed, we demonstrate through the spectral analysis that the error compensation characteristic of motor synergies occurs only at low frequencies, whereas the fingers interact with each other synchronously at higher frequencies. We found that the deprivation of one sensory modality results in increased sensory delays and latencies, which needs to be compensated by the other sensory modalities. We conclude that the neurophysiological structures similar to the system of Renshaw cells involving short-latency feedback and back-coupling gain (that cannot be measured directly through experiments) can explain the deterioration of synergy invoked by sensory deprivation.

## Results

**Figure 1:**
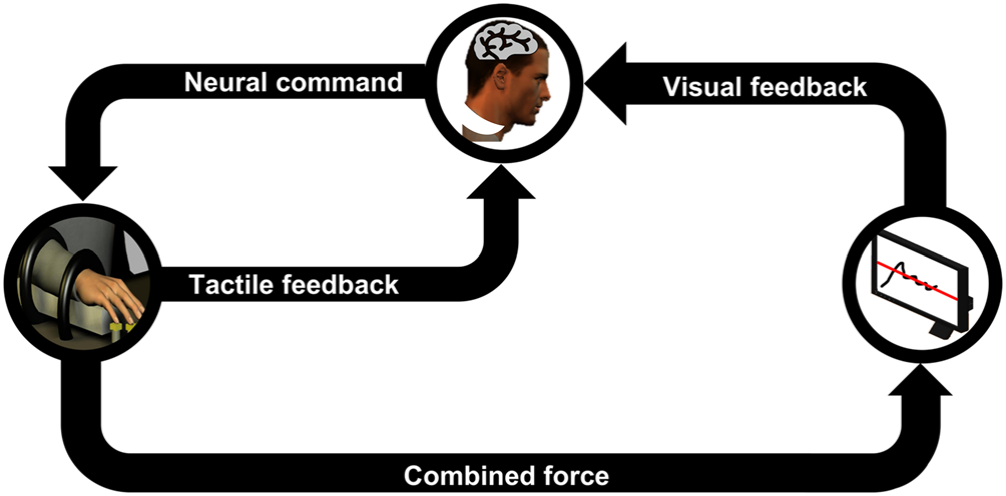
Diagram of the finger force production model. The subject is tasked with producing a constant force by pressing their four fingers (index, middle, ring, and little) on force sensors in two conditions: with and without tactile feedback. The force sensors record the produced force at each finger. The combined force of all fingers (as a black line), as well as the target line (as a red line), are provided as visual feedback to the subject using a display. The CNS integrates the information gathered from visual and tactile feedback to provide the required neural command to the fingers for matching the total force with the target force. The resulting performance is validated against human-generated data of a finger force production experiment from a previous study^17^.

In this section, we propose a model to explain some of the neuromechanical characteristics of the human sensorimotor system. This model was initially inspired by the finger force production experiment performed in our previous study^17^ but may be extended to other tasks. We later validate the results of our model using those experimental data. In that experiment, we asked right-handed participants (n = 18) to use the four fingers of their right hand and press on force sensors to produce a constant force of 20 N. A computer screen displays the sum of the forces generated by all fingers along with the 20 N target force (Fig. 1). They performed the task under two conditions: with and without tactile feedback (achieved by digital anesthesia). For each condition, they repeated the task for 12 trials, each 12 s in duration. Experimental details can be found elsewhere^17^.

### Proposed model

**Figure 2:**
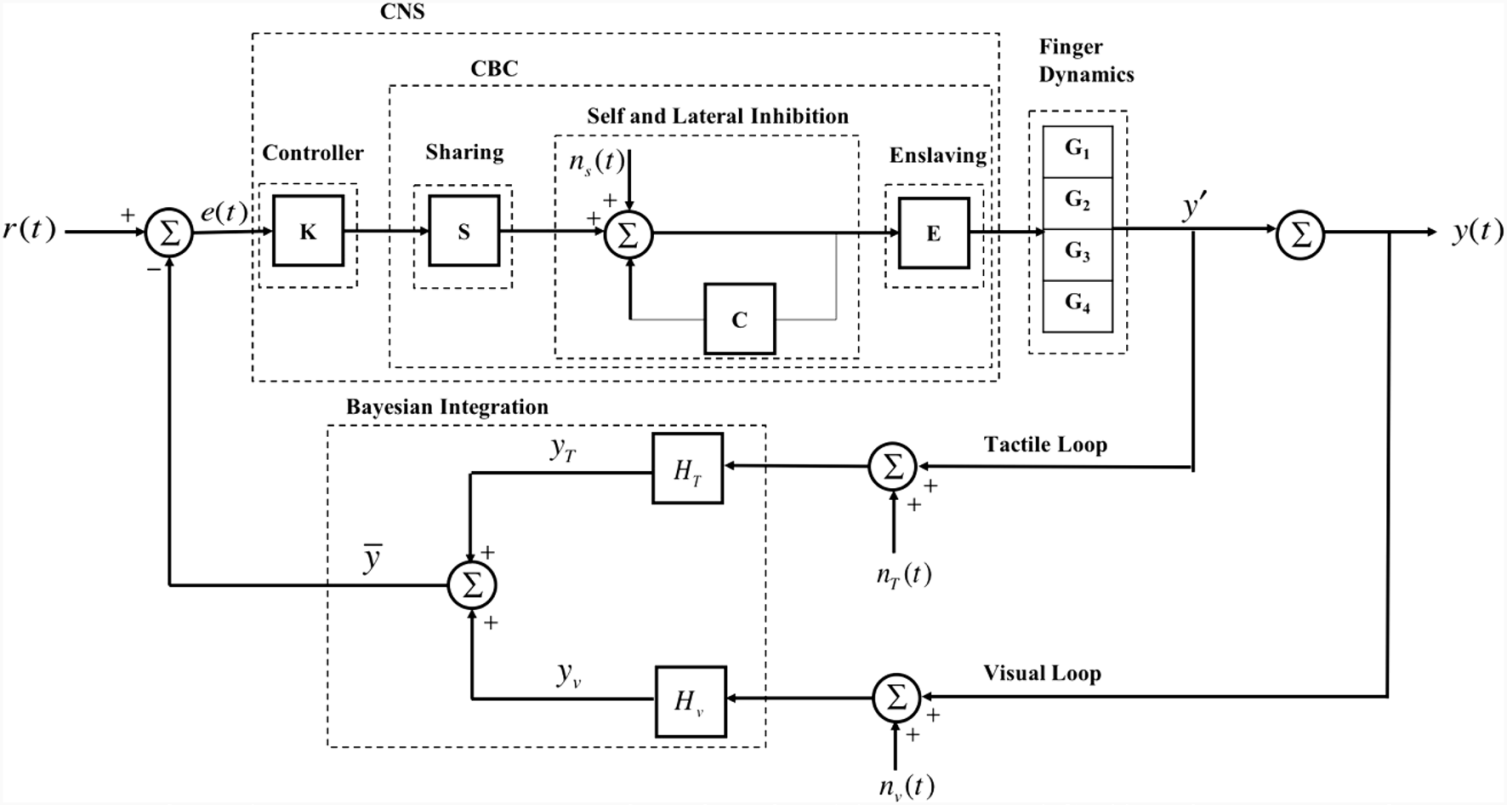
Schematic of the proposed model for finger force production task. The system has the desired force, *r*(*t*), as input and, three noises or disturbances: *n*_*s*_(*t*) for sharing noise, *n*_*T*_(*t*) and *n*_*v*_(*t*) for tactile loop and visual loop noises, respectively, and an output, *y*(*t*), i.e., sum of four finger forces. The controller, K, is a PD-lag controller that produces a command that minimizes the difference between *r*(*t*) and *y*(*t*). Sharing (S) defines the share of force that each finger needs to contribute to satisfy the total command by the controller. Self and lateral inhibition accounts for neural connection between the fingers and forms the finger synergistic interaction using a unity forward-path gain and a negative feedback-loop gain with transport delay (C). Enslaving (E) accounts for the mechanical interconnection between fingers. K, S, self and lateral inhibition and E represent the CNS in our model. Each finger dynamics (*G*_*i*,_ $i \in \{ 1,2,3,4\}$) is modeled as a second-order linear system. Sum of forces produced at each finger yields the total force. Tactile loop and visual loop simulate the tactile (from the four finger force sensors) and visual (from eyes observing the total force on screen) sensors, respectively, which are integrated using Bayesian rule and fed back to K.

Here we provide a mathematical model for the synergistic interaction between motor effectors within the human sensorimotor system. Using this model, we simulate the finger forces required for performing the task explained in the experiment mentioned above. The model consists of a controller to perform the role of the CNS, a plant that captures finger dynamics, and sensors for including the tactile and visual feedback mechanisms (Fig. 2).

The mathematical model captures different structures in the human sensorimotor system. We capture the multiple roles of the CNS as follows: First, a PD-lag compensator is used to represent the control role of CNS. Second, consistent with motor synergy theory and CBC hypothesis^24^, we add sharing, self and lateral inhibition, and enslaving to capture the synergetic aspect of the sensorimotor system. We use a second-order linear system to represent the dynamic response of individual fingers^26^.

**Figure 3:**
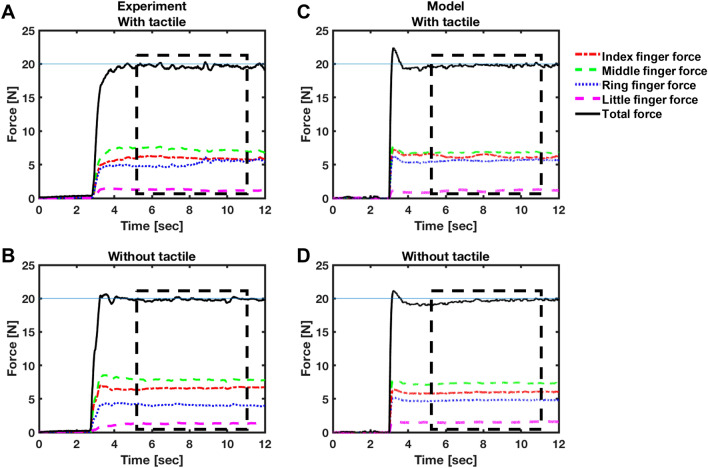
Experimental and simulated finger force trajectories for two trials. Human-subject experimentally-generated finger forces in (**A**) with tactile and (**B**) without tactile feedback, respectively. Simulated finger force trajectories for randomly generated human-subject parameters (**C**) with tactile and (**D**) without tactile feedback. The black line shows the sum of all finger forces. Each finger force is shown with a designated color. The dashed line shows the 5 s window where the total force remains stationary, which is the part of data for our HVD analysis. We use this part of data for our analysis.

The CNS compares the desired and total sensed multi-digit forces and determines the compensatory forces that need to be produced by each finger to reduce the error between desired and actual multi-digit finger forces. The fingers then produce these requisite forces, and the resultant multi-digit force is obtained by adding up the individual finger forces. The tactile sensors in the fingers measure individual finger forces, while the visual sensors (eyes) measure the resultant force. These sensor measurements are finally integrated using Bayesian multi-sensory integration^6,7^ and fed back to the CNS. The simulated and experimental finger force trajectories are shown in Fig. 3. The mathematical representation of different structures of the model and the parameters chosen for simulating finger forces have been explained extensively in the “Materials and methods” section.

### Spectral analysis of experimental data

The examination of individual finger forces indicates the existence of drift over time in the experimental force trajectories (Fig. 3A and B). To better understand the mechanism behind the drift component observed in empirical data, spectral analysis was performed. Frequency domain measures (spectral analysis) have identified the existence of in-phase and anti-phase patterns of coordination between each pair of fingers at different frequencies. In particular, the complex coherence^27^—a complex-valued function of frequency that characterizes the linear relationship between two signals—between each pair of finger forces (index-middle, index-ring, etc.) is calculated (for more details refer to “Materials and methods” section).

**Figure 4:**
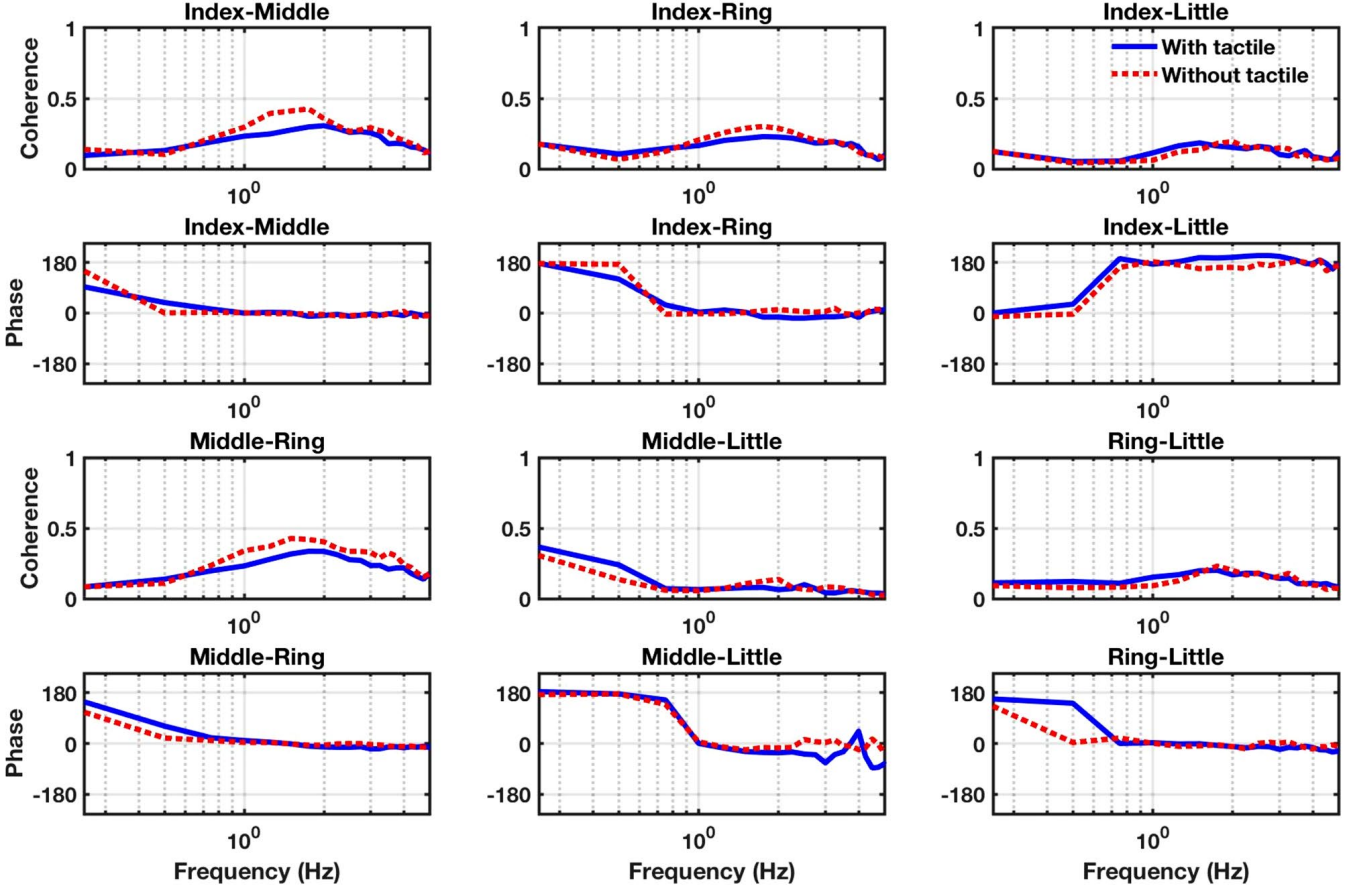
Complex coherence. Squared magnitude and phase of coherence for combinations of finger forces (index-middle, index-ring, index-little, middle-ring, middle-little, and ring-little) are shown. Blue and red lines correspond to tactile and no tactile feedback conditions, respectively.

Figure 4 represents the squared magnitude and phase of complex coherence for the duration of the trial that the total force was nearly stationary (5–11 s). As Fig. 4 demonstrates, for all the pairs except for the index-little pair, the signals are anti-phase at low frequencies (below 1 Hz). As we move to higher frequencies (1.6 Hz–2.5 Hz), the coherence becomes higher (around 0.4), and the signals become in-phase (i.e., phase shift between the two signals is almost zero). On the other hand, the index-little finger pair reveals an opposite trend to the other finger pairs (i.e., out-phase in low frequencies and in-phase at high frequencies). The in-phase and out-phase behavior can be traced back to the positive and negative covariation between the two signals, respectively. A negative covariation between the two signals is an indication of error compensation between fingers^17^, providing evidence for the existence of synergy at low frequencies.

### Validation of the proposed model using experimental data

In order to examine if our model can capture the general human behavior after sensory deprivation, we compare our simulated finger force trajectories with the previously collected experimental data^17^ using HVD analysis^17^. This analytical technique provides a linear decomposition of variability in the motor output of redundant tasks. Within the HVD framework, the task performance error is quantified as the overall mean-squared error (OMSE), defined as the sum of squared deviations from the task goal. OMSE can be further decomposed to three error components as performance variables: 1) The online variable error $(\overline{{Var(\tilde{y})}} )$, defined as the variance within a trial, averaged over the trials, 2) The offline variable error ($Var(\varepsilon )$), defined as the variance between trials, and 3) the systematic error ((*r-m*)^2^), defined as the squared deviation between target force (i.e., *r*(*t*) = 20) and *m*, where *m* is the mean total force after averaging over all timesteps of all 12 trials. Throughout the remainder of this work, online control refers to moment-to-moment (i.e., within a trial) control, and offline control refers to trial-to-trial (i.e., between trial) control.

Using the hierarchical structure of variability, the online and offline errors can be further partitioned into the sum of individual finger force variances (i.e., $\sum\limits_{k = 1}^{4} {Var(\tilde{f}_{k} )}$ for online, and $\sum\limits_{k = 1}^{4} {Var(\varepsilon_{k} )}$ for offline), plus the sum of between-finger force covariances (i.e., $\sum\limits_{j \ne k}^{4} {Cov(\tilde{f}_{j} ,\tilde{f}_{k} )}$ for online, and $\sum\limits_{j \ne k}^{4} {Cov(\varepsilon_{j} ,\varepsilon_{k} )}$ for offline). The negation of covariance is mathematically equivalent to the index of synergy^17^.

**Figure 5:**
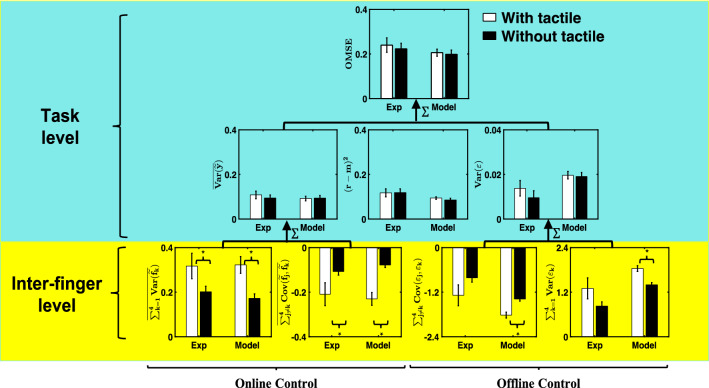
The HVD analysis for the experiment and simulation. The white and black bars show with and without tactile feedback conditions. At task level, the OMSE is equal to the sum of online variance ($\overline{{Var(\tilde{y})}}$, offline variance ($Var(\varepsilon )$) and systematic error ((*r-m*)^2^). At inter-finger level, shown in yellow, the online and offline variances can be further partitioned into the sum of individual finger force variances (i.e., $\sum\limits_{k = 1}^{4} {Var(\tilde{f}_{k} )}$ for online, and $\sum\limits_{k = 1}^{4} {Var(\varepsilon_{k} )}$ for offline), plus the sum of between finger force covariances (i.e., $\sum\limits_{j \ne k}^{4} {Cov(\tilde{f}_{j} ,\tilde{f}_{k} )}$ for online, and $\sum\limits_{j \ne k}^{4} {Cov(\varepsilon_{j} ,\varepsilon_{k} )}$ for offline). The negation of covariance is mathematically equal to the index of synergy. Both model and experiment demonstrate significant differences between the two tactile conditions in $\overline{{\sum\limits_{k = 1}^{4} {Var(\tilde{f}_{k} )} }}$ and $\sum\limits_{j \ne k}^{4} {Cov(\tilde{f}_{j} ,\tilde{f}_{k} )}$. The model shows differences between two conditions in $\sum\limits_{k = 1}^{4} {Var(\varepsilon_{k} )}$ as well. The error bars for experiment and model represent the standard error across participants and runs of simulations (216 times = number of subject*number of trials), respectively.

In Fig. 5, the comparison between the result of the simulation and experiment using HVD analysis is presented. To examine whether our model can capture the experimental behavior before and after the removal of tactile feedback, we performed statistical analysis (i.e., t-test with the significance value of 0.05) on all the variables in the HVD to compare the two tactile conditions of our simulation. The results of these t-tests were compared to their experimental counterparts^17^.

In all levels of the hierarchy except for $\sum\limits_{k = 1}^{4} {Var(\varepsilon_{k} )}$ (t(17) = − 22.08, *p* = 5.9016e-14) and $\sum\limits_{j \ne k}^{4} {Cov(\varepsilon_{j} ,\varepsilon_{k} )}$ (t(17) = − 22.04, *p* = 6.0636e-14), simulation results could reproduce the experiment in terms of the statistical analysis. In particular, from the result of simulation, we found that at the highest level of hierarchy for the model, OMSE (t(17) = 1.19, *p* = 0.25), $\overline{{Var(\tilde{y})}}$(t(17) = − 1.74, *p* = 0.1), $Var(\varepsilon )$(t(17) = 0.45, *p* = 0.66), and systematic error (t(17) = 1.99, *p* = 0.06) remained statistically unchanged after removal of the tactile loop. This is consistent with experimental results^17^.

Moreover, again in line with our previous experiment^17^, the online variance and covariance significantly decreased after removal of tactile feedback ($\overline{{\sum\limits_{k = 1}^{4} {Var(\tilde{f}_{k} )} }}$ (t(17) = 8.32, *p* = 2.1205e-07) and $\sum\limits_{j \ne k}^{4} {Cov(\tilde{f}_{j} ,\tilde{f}_{k} )}$ (t(17) = − 8.17, *p* = 2.7082e-07). These results indicate that the model is capable of reproducing the synergistic interaction between fingers with and without tactile feedback, confirming the results that the interactions between fingers at the lower-level change such that they compensate for the degradation in tactile sensory feedback and maintain the performance at the higher level.

### The effect of sensory deprivation on different components of the model

In this section, we explain how we addressed the main questions of the paper and summarize the results of our different simulations.

First, to investigate how the lack of one sensory modality affects sensory delays and latencies, we tested two hypotheses regarding the impact of anesthesia on tactile feedback. In one case, we hypothesized that anesthesia would completely remove the tactile feedback, and in another case, we hypothesized that anesthesia would just attenuate tactile feedback. So, we removed the tactile loop from the model in the former case, while reduced the tactile gain in Bayesian sensory integration for the latter case. In the former case, the simulation resulted in reduced OMSE in without tactile feedback condition (t(17) = 7.80, *p* = 5.15e-07), which contradicted the unchanged OMSE between the two conditions that was observed in the experiment (see supplementary Fig. S1). In the latter case, the model adequately reproduced the trend in OMSE (t(17) = − 1.56, *p* = 0.14) but not the online individual finger variances and online synergy (see supplementary Fig. S2). Particularly, this simulation resulted in unchanged online individual finger variance ((t(17) = 0.94, *p* = 0.36)) and online synergy ((t(17) = − 1.60, *p* = 0.13)). So, since removing the tactile loop in the first hypothesis failed to reproduce the trend for OMSE, in line with the second hypothesis, we decided to keep the tactile loop in the model and decrease the gain associated with tactile feedback to at least maintain the level of OMSE.

Second, we investigated different components of the model to address the issue with the online synergy. Specifically, to examine which aspects of sensorimotor system are responsible for the reduced online synergy after sensory deprivation in experiment, according to Eq. (15) in the “Materials and methods” section, we postulated two hypotheses. The first hypothesis was that the CNS changes its neural commands to the motor effectors when it faces sensory deprivation. To test this hypothesis, we designed different controllers for different tactile conditions, and were able to reproduce the reduction in synergy due to sensory deprivation. The second hypothesis was that changes in different components of the CBC model will affect the synergy. The conjecture was based on previous studies, where it was shown that anesthesia would not impact enslaving and sharing. Plus, CBC model^24^ postulated that back coupling loops are responsible for creating the between finger synergy. To test this hypothesis, we changed the gain and delay associated with CBC to see their effect on synergy. We found that by decreasing the CBC gain and increasing the CBC delay, we are able to reproduce the reduction in online synergy due to anesthesia (Fig. 5). Therefore, both hypotheses led to recreation of the trend between the two tactile conditions. In the next section, we discuss which hypothesis can better explain the mechanism of the reduction in online synergy after removal of tactile feedback. We also observed that by removing the CBC from the model, no differences between the two conditions were observed, plus the covariance term becomes positive (see supplementary Fig. S3).

## Discussion

We investigate the underlying neuromechanical mechanisms behind the synergetic behavior of redundant motor control by providing a mathematical model for a multi-digit finger force production task. The model presented in this paper simulates the finger force production task under two tactile conditions: with and without tactile feedback. As it is shown in Fig. 5, the proposed model is able to reproduce the synergistic interactions between fingers similar to the experiment under both tactile conditions^17^. In previous work^17^, we provided analysis showing that tactile sensors play a substantial role in the synergistic interactions between fingers. We found that although deprivation of tactile sensation degrades the synergistic interaction between fingers, it does not affect the performance error^17^.

In this paper, we mainly identify the underlying neural mechanism behind this phenomenon using a gray box model. The presented model is consistent with known biological characteristics. First, the CNS integrates the sensory information to plan motion and produce motor commands to accomplish a required task. Second, the tactile information is transmitted to the CNS via the peripheral (median, ulnar, and radial) nerves at each finger, and integrated with the visual information that is transmitted to the CNS directly. Our model demonstrates that the inherently noisy and delayed sensory systems can be integrated according to the Bayesian rule and fed back to CNS to determine appropriate commands to the motor system and ensure synergistic interaction between effectors. Thus, sensory and motor systems can be integrated and interplayed in a closed-loop structure to perform a task. A thorough investigation of our model and experimental data provides important implications regarding synergy and the role of tactile feedback on synergy, which will be discussed in the following paragraphs.

### Error compensation of synergies occurs only at low frequencies

We observed a counter-directional drift in the finger forces trajectories (Fig. 3) that allowed the total force to remain close to the target force after about 5 s. In other words, if one finger deviates from its share of force and causes an error in the total force produced by all the fingers, the other fingers compensate for that error to maintain the total force. Previous studies demonstrated the existence of unintentional drop in total force after the removal of visual feedback in the task of multi-finger force production, which would lead to counter directional drift in individual finger forces, i.e., drift toward higher or lower forces^28^. The origin of the drop in total force was mentioned to be the natural tendency of the physical system to move toward the stable state associated with minimum potential energy^28^. As in all conditions, participants had access to visual feedback, yet we did not observe a drift in total force. However, we observed the counter directional changes in individual finger forces even in the presence of visual feedback. In fact, some fingers drift toward higher forces, and some drift toward lower forces to maintain the total force. This feature refers to the error compensation property of motor synergies. Our spectral analysis demonstrates that the compensatory feature occurs only at low frequencies, and the finger forces become synchronous at higher frequencies. It is worth noting that although the error compensation between fingers decreased due to anesthesia, it did not completely disappear. The effect of anesthesia on synergy will be discussed later in this section.

Interestingly, our spectral analysis revealed that index-little finger forces switched from an in-phase pattern at low frequencies to an anti-phase pattern at higher frequencies, the opposite pattern as the other finger pairs (Fig. 4). This spectral phase switch could be related to the mechanical coupling of the index and little fingers and traced back to the enslaving effect or muscle activation^29^. For example, a previous study proposed that in a multi-finger prehension task that requires fingers to produce higher forces for holding a large object, the antagonist muscles of peripheral fingers (i.e., index and little fingers) need to be activated to prevent slipping of the object^30^. The role of mechanical coupling in spectral phase shifts is better understood in other human motor behaviors, such as standing balance, where the kinematics of segment angles exhibit spectral phase switches but the electromyographic (EMG) activities of muscles do not^31^. A fuller understanding of the spectral phase shifts of finger pairs, a novel finding of our study, would be aided by future studies that similarly included both kinetic and EMG measurements.

In addition, from our observation, we concluded that the sharing function also has a drifting trend. The previous studies^5,32^ had suggested that the sharing ratios remains constant during a trial, but the results of this paper show that sharing patterns have a drifting trend. Thus, we cannot use predefined fixed values for reproducing synergistic interaction between fingers; if we were to use fixed sharing patterns, we would not be able to reproduce the online and offline variance and covariance profiles as well as OMSE (see supplementary Fig. S4). We incorporated the pink noise (known to be responsible for coordination between effectors^33,34^) as well as a lag compensator to account for the drift in the behavior.

### The implications of the proposed neuromechanical model for sensorimotor system

Given the successful task accomplishment by the subjects under both tactile sensation conditions in maintaining the same level of OMSE, two possible hypotheses were postulated concerning the changes invoked by anesthesia. First, the tactile sensation is completely gone, and therefore, we need to remove the tactile loop from our model. An alternative hypothesis would be keeping the tactile loop and reducing the tactile gain in Bayesian sensory integration to reduce the reliability of tactile feedback due to anesthesia.

Removing the tactile loop means that participants solely depend on the remaining sensory feedback, particularly the visual feedback, to accomplish the task. This assumption seems reasonable, as the task performed is visually dominant. Besides, in general, we tend to rely more on our vision to accomplish tasks^35^. However, upon testing this hypothesis, we find that removing the tactile loop would lead to less OMSE in without tactile feedback condition, as the noises associated with the tactile loop would also be removed. However, the experimental results demonstrated unchanged OMSE in both conditions.

A recent study suggested that force control in gripping can be preserved even if the somatosensory information is perturbed through anesthesia^14^. They declared that residual sensory input within the hand/forearm could be responsible for this phenomenon. Therefore, the preserved OMSE, $\overline{{Var(\tilde{y})}}$, $Var(\varepsilon )$, and systematic error in both conditions in the experiment can be partially explained by the residual sensory input within the fingers. So, we tested our second hypothesis and reduced the tactile gain in the Bayesian sensory integration^6,7^ to account for the tactile sensory deprivation. Hence, according to the Bayesian multi-sensory integration principle, the tactile sensation will be less reliable due to anesthesia, and participants have to rely more on their vision. However, this is only part of the story. Our model confirms previous findings that tactile sensory deprivation results in longer sensory transduction delays, and increased latencies^8,10,13^. So, to account for longer response time and delays, we increased the tactile sensory delay. While the results in preservation of the OMSE levels observed empirically, the decrease in online synergy due to anesthesia-observed empirically- was not reproduced by this change (see supplementary Fig. S2).

To understand the underlying neural mechanism behind the degradation of synergy, we scrutinize different components of our model. Previous studies have declared that anesthesia does not change the enslaving matrix and sharing^14^. So, given the Eq. (15) in the “Materials and methods” section, we can pose two additional hypotheses. The first hypothesis is that the controller is different between conditions. From the control-theoretic point of view, one can of course redesign a new controller for new specifications that give the intended results if within the dynamical system’s capabilities. Our model was not an exception to this fact, either. In other words, we designed a new controller for without tactile feedback case, and we could reproduce the experimental HVD.

Nevertheless, we are interested in determining if a different mechanism is responsible for the synergy changes to the sensorimotor system. Does the neural command change due to loss of sensation? If so, what part of the CNS is responsible for it? This is a non-trivial question which leads us to the second hypothesis. Given no observed changes in sharing and enslaving patterns between the two conditions experimentally, and given Eq. (15) in the “Materials and methods” section, we hypothesized that the back-coupling gain and delay must be different between the two conditions to achieve the change in synergy.

In the CBC scheme, the short-latency negative feedback loops can mimic the action of synergy without any need for sensory feedback^24^. The inhibitory connections between the elements within this scheme demonstrate error compensation between elements. In other words, the elements act in a way that an increase in the activity of one would lead to a decrease in the activity of others such that the error in the performance would be minimal. This kind of error compensation between the elements eliminates the need for sensory feedback^24^. Similarly, another study^20^ proposed that compensation can be observed in a pure feedforward control scheme as long as the controller has information about the relationship between the change in the output to the changes in the individual elements (i.e., the Jacobian of the system).

Our model confirms the CBC hypothesis that self and lateral inhibitions play an essential role in producing the synergistic interaction between motor effectors. However, our model provides evidence that contrary to the CBC hypothesis, these back-coupling loops are not independent of the sensory feedback in the sensorimotor system. In other words, the back-coupling loops are not sufficient for reproducing the changes in synergistic interaction between fingers if the sensory feedback is manipulated (see supplementary Fig. S3). Our model demonstrates that the gain and delay of the back-coupling loops are associated with the manipulation of sensory feedback. In particular, anesthesia resulted in lower gain and higher delay. Thus, changes to gain and delay can further explain the reason behind the reduction in synergy in response to sensory deprivation. There are no experimental ways to measure the back-coupling loop gain, but, it has been suggested this gain is tunable^24^. Our model provides a means to test this hypothesis without a need for further experiments. Changing the back-coupling loop gain and delay in the without tactile feedback condition does in fact lead to the reproduction of the experimental HVD results.

Although from both hypotheses, we could reproduce experimental HVD results, the second hypothesis is more reasonable as it is in line with the hierarchical structure of motor control. In particular, the unchanged performance error in both conditions and decreased online variance in experimental HVD results suggests that CNS responds to anesthesia and sensory deprivation by reducing the variability at the lower level and without the need to change the overall behavior at task level.

This model further suggests that the derived feedback control model of sharing, self and lateral inhibition, enslaving, and Bayesian multi-sensory integration, all together, account for the essential components of the CNS control strategy for the control of redundant tasks even after removal of one sensory modality. This result confirms the principle of minimal intervention^2^ and the critical role of sensory feedback on the formation of these variability patterns. This theory states that CNS uses feedback to correct deviations that interfere with the task goal but allows variability in redundant dimensions.

Our model not only can serve as a proxy for understanding the neuromechanical mechanism of synergistic interaction within the sensorimotor system, but it can have many clinical and industrial applications. Particularly, our better understanding of the sensorimotor system can aid in the design of collaborative robots. One way to achieve a high level of action recognition from robots is to first discern the underlying neural mechanism behind the human sensorimotor system. Our analytical control designs can be used to generate human-like responses for collaborative robots that feel intuitive and natural. Furthermore, using our model, we can test hypotheses that could not be performed via real experiments. Future work would consider the effects of removing the visual loop from the model and assess performance changes without the need for human trials. Thus, our ultimate goal of modeling this sensorimotor system is to reveal potential systematic mechanisms employed by the CNS to control our movement, and to leverage garnered insights for clinical applications and intuitive control design of robots.

We intend to provide a simple high-level representation of the sensorimotor system that can explain the unknown gaps in our understanding of the association between the sensory system and the synergistic interaction between effectors. Still lacking from our model is the ability to explain the trial-to-trial variance and synergy. Even though the undetected change between two conditions can be due to the high standard error and the between-subject variability, this issue needs to be further investigated in future studies.

## Materials and methods

In this section, a brief description of the human-generated finger force experiment^17^ is provided for completeness, as well as a thorough explanation of the proposed mathematical model. We use the experimental data to validate the proposed model for investigating the underlying neural mechanism of the sensorimotor system. Full details of participants and the experimental setup can be found in a previous study^17^. The study was approved by the ethics committee of Korea University. All the procedures were carried out in accordance with the relevant guidelines. Informed consent was obtained from each participant.

1. *Participants:* The experimental data analyzed here were collected from eighteen healthy right-handed male participants (average age of 23.95 ± 1.00 years) with no history of neurological disorders.
2. *Experimental setup:* Participants were asked to rest each of the four fingers (i.e., index, middle, ring, and little) of their right hand on force sensors such that they matched the target force shown on the screen. The subject was shown the visual feedback of the combined force produced by the fingers along with the target force in the form of a red horizontal line on a computer screen (Fig. 1). Each participant performed the task under two conditions: with and without tactile feedback. Each condition consisted of 12 trials, in each of which the subject was asked to produce a constant force of 20 N (total endpoint fingertip forces, summed over all four fingers) over 12 s. In the without tactile condition, which was performed on a separate day, local anesthetic was injected into fingers.

### Mathematical model design: multi-digit finger force production

The proposed feedback model consists of a controller to perform the control role of CNS, a plant that captures finger dynamics and sensors for including the tactile and visual feedback mechanisms (Fig. 2).

The CNS has multiple roles that we attempt to capture in our model. A PD-Lag controller is used to resemble the control role of the CNS, where the proportional component explains how CNS controls the response time of the system as it changes the force from zero to the target force. The derivative component explains the ability of the CNS to anticipate performance changes and react accordingly. This part is in support of what forward model theory^36^ proposes, that given the knowledge of the current state of the system (i.e., position and velocity) as an input, the controller sends commands in a way that anticipates the future state of the system. Finally, the lag component allows us to incorporate the observed empirical drift present in the produced force. This component explains how the CNS handles outdated information^3^. Similar to our previous work^24^, to capture the synergetic aspect of the sensorimotor system, Sharing^5^, Enslaving^22^, and Self and Lateral Inhibitions^24^ are included in the model. These plants are modeled consistent with the motor synergy theory and CBC model.

The CNS compares the desired versus sensed multi-digit forces at a higher level and determines the forces required to be produced by each finger. The fingers then produce these requisite forces, and the resultant multi-digit force is obtained by adding up the individual finger forces. The tactile sensors in the fingers measure individual finger forces, while the visual sensors (eyes) measure the resultant force. These visual and tactile sensor measurements are finally integrated and fed back to the CNS, which then determines the compensatory finger forces based on motor synergy theory and CBC model^24^ to eliminate the error between desired and actual multi-digit finger forces.

The sharing function dictates how the resultant compensatory finger force (*u*_*c*_) should be allocated to each finger. Force sharing characteristic is one example of the redundancy problem where different combinations of force can produce the same total force. Denoting the output of the sharing function as the reference force to be produced at each finger $f_{k}^{REF}$ (*k* = 1, 2, 3, 4 where 1, 2, 3 and 4 indicate index, middle, ring, and little fingers), the sharing function is modeled as a vector of constants:

1 $$F^{REF} = \left[ {\begin{array}{*{20}l} {f_{1}^{REF} } \hfill \\ {f_{2}^{REF} } \hfill \\ {f_{3}^{REF} } \hfill \\ {f_{4}^{REF} } \hfill \\ \end{array} } \right] = Su_{c} = \left[ {\begin{array}{*{20}l} {s_{1} } \hfill \\ {s_{2} } \hfill \\ {s_{3} } \hfill \\ {s_{4} } \hfill \\ \end{array} } \right]u_{c}$$

where *s*_*i*_ > 0 for $i \in \{ 1, \ldots ,4\}$ and $\sum\limits_{i = 1}^{4} {s_{i} = 1}$.

The lateral inhibition or CBC function represents synergistic behaviors that are common in neuro-physiological systems^37^, such as auto- and cross-inhibition of finger forces and interaction delays occurring in multi-digit coordination tasks. The model developed in^24^ is used in this study, which is a feedback system consisting of a unity forward-loop gain and a negative feedback-loop gain ($\gamma_{c}$) with transport delay.

2 $$Z = \left[ \begin{gathered} z_{1} \hfill \\ z_{2} \hfill \\ z_{3} \hfill \\ z_{4} \hfill \\ \end{gathered} \right] = (I_{4 \times 4} - e^{{ - \tau_{CBC} s}} \gamma_{c} )^{ - 1} F^{REF} = \left( {I_{4 \times 4} - e^{{ - \tau_{CBC} s}} \left[ {\begin{array}{*{20}c} {g_{11} } & \cdots & {g_{14} } \\ \vdots & \ddots & \vdots \\ {g_{41} } & \cdots & {g_{44} } \\ \end{array} } \right]} \right)^{ - 1} F^{REF}$$

where *Z* is the output of CBC, $I_{4 \times 4}$ is a four-by-four identity matrix, *s* is the Laplace variable, $\tau_{CBC}$ is the transport delay associated with the neural pathways, and *g*_*ij*_ ($1 \le i,j \le 4$) denotes the inhibitory interaction between fingers *i* and *j*.

The enslaving function accounts for the fact that activation of a finger elicits co-activation of other fingers due to the mechanical coupling from the physical interconnection between them^22^. This function is modeled as a constant matrix gain (*E*):

3 $$F^{SS} = \left[ \begin{gathered} f_{1}^{SS} \hfill \\ f_{2}^{SS} \hfill \\ f_{3}^{SS} \hfill \\ f_{4}^{SS} \hfill \\ \end{gathered} \right] = EZ = \left[ {\begin{array}{*{20}c} {e_{11} } & \cdots & {e_{14} } \\ \vdots & \ddots & \vdots \\ {e_{41} } & \cdots & {e_{44} } \\ \end{array} } \right]Z$$

where the output of the enslaving function *F*^*SS*^ denotes the reference steady-state force at the individual fingers.

To describe the dynamic behavior of the fingers, we assumed a second-order linear system (i.e., a mass-spring-damper system). We consider digit motion only along one direction and assume a linear damped second-order model (one degree-of-freedom)^26^:

4 $$F = \left[ {\begin{array}{*{20}l} {f_{1} } \hfill \\ {f_{2} } \hfill \\ {f_{3} } \hfill \\ {f_{4} } \hfill \\ \end{array} } \right] = G(s)F^{SS} = \left[ {\begin{array}{*{20}l} {G_{1} (s)} \hfill & 0 \hfill & 0 \hfill & 0 \hfill \\ 0 \hfill & {G_{2} (s)} \hfill & 0 \hfill & 0 \hfill \\ 0 \hfill & 0 \hfill & {G_{3} (s)} \hfill & 0 \hfill \\ 0 \hfill & 0 \hfill & 0 \hfill & {G_{4} (s)} \hfill \\ \end{array} } \right]F^{SS}$$

where *F* is the vector of individual finger forces, $G_{k} (s) = \gamma_{k} \omega_{n,k}^{2} /(s^{2} + 2\zeta_{k} \omega_{n,k}^{2} + \omega_{n,k}^{2} )$, and, $\gamma_{k}$, $\zeta_{k}$, and $\omega_{n,k}$ are gain, damping ratio and natural frequency associated with (*G*_*k*_ (*s*)*, k* = 1, 2, 3, 4).

In summary, the control command *u*_*c*_ from the CNS is related to the individual finger forces *F* as follows:

5 $$F(s) = G(s)E(I_{4 \times 4} - e^{{ - \tau CBC^{S} }} \Gamma )^{ - 1} Su_{c} (s)$$

where *F*(*s*) and *u*_*c*_(*s*) are explicitly expressed as functions of *s* for clarity.

The sensory feedback signal transmitted to the CNS $\overline{y\left( t \right)}$ is modeled as the Bayesian integration of visual and tactile feedback signals as shown in Eq. (6)

6 $$\overline{y\left( t \right)} = B\left( {y_{v} \left( t \right),y_{T} \left( t \right)} \right) = \frac{{{\raise0.7ex\hbox{$1$} \!\mathord{\left/ {\vphantom {1 {\sigma_{V}^{2} }}}\right.\kern-\nulldelimiterspace} \!\lower0.7ex\hbox{${\sigma_{V}^{2} }$}}}}{{{\raise0.7ex\hbox{$1$} \!\mathord{\left/ {\vphantom {1 {\sigma_{V}^{2} }}}\right.\kern-\nulldelimiterspace} \!\lower0.7ex\hbox{${\sigma_{V}^{2} }$}} + {\raise0.7ex\hbox{$1$} \!\mathord{\left/ {\vphantom {1 {\sigma_{T}^{2} }}}\right.\kern-\nulldelimiterspace} \!\lower0.7ex\hbox{${\sigma_{T}^{2} }$}}}}y_{V} \left( t \right) + \frac{{{\raise0.7ex\hbox{$1$} \!\mathord{\left/ {\vphantom {1 {\sigma_{T}^{2} }}}\right.\kern-\nulldelimiterspace} \!\lower0.7ex\hbox{${\sigma_{T}^{2} }$}}}}{{{\raise0.7ex\hbox{$1$} \!\mathord{\left/ {\vphantom {1 {\sigma_{V}^{2} }}}\right.\kern-\nulldelimiterspace} \!\lower0.7ex\hbox{${\sigma_{V}^{2} }$}} + {\raise0.7ex\hbox{$1$} \!\mathord{\left/ {\vphantom {1 {\sigma_{T}^{2} }}}\right.\kern-\nulldelimiterspace} \!\lower0.7ex\hbox{${\sigma_{T}^{2} }$}}}}y_{T} \left( t \right)$$

where *B*(*.*) is the Bayesian sensory fusion function, while *y*_*v*_(*t*) and *y*_*T*_(*t*) are visual and tactile sensory signals, respectively. As shown in Fig. 2, the visual sensory system measures the resultant multi-digit finger force as displayed on the screen while the tactile sensory system measures the forces produced at the individual finger level. Noting that both sensory feedback mechanisms involve measurement noise and transport delays along their neural pathways^9^, and also that the CNS applies the Bayesian sensory fusion to the resultant multi-digit finger forces to determine the compensatory force *u*_*c*_, *y*_*v*_(*t*) and *y*_*T*_(*t*) can be written as follows:

7 $$\begin{aligned} & y_{V} (t) = \left[ {\sum\limits_{k = 1}^{4} {f_{k} (t - \tau_{V} )} } \right] + n_{V} (t) \\ & y_{T} (t) = \left[ {\sum\limits_{k = 1}^{4} {f_{k} (t - \tau_{T} )} } \right] + n_{T,k} (t) \\ \end{aligned}$$

where $\tau_{V}$ and $\tau_{T}$ are the transport delays associated with visual and tactile sensory pathways, and $n_{V} \sim N(m_{V} ,\sigma_{V}^{2} )$ and $n_{T,k} \sim N(m_{T} ,\sigma_{T}^{2} )$ are the normally distributed disturbances associated with visual and tactile sensory systems, where *m* and $\sigma$ denote the mean and standard deviation of the noise distributions. The input to the CNS (*e*(*t*)) is given by the error between the reference (*r*(*t*); modeled as a step function with final value of 20 N to simulate the horizontal bar target force displayed on the screen in the experiment) versus Bayesian-integrated multi-digit finger force signals (Fig. 2):

8 $$e(t) = r(t) - y(t) = r(t) - {\text{B}}(y_{V} (t),y_{T} (t))$$

### Simulation

To simulate the multi-digit finger force production task using the mathematical model described in the previous section, we choose the parameters in the model as follows. First, to determine the sharing ratio, for each trial, the data from 5 to 11 s of the with tactile feedback condition is extracted. This part is selected to avoid the transient force stabilization at the beginning of each trial and premature cessation of force production at the end^32^. Then, the experimental sharing parameters *s*_*k*_ (*k* = 1, 2, 3, 4) corresponding to each trial are determined as the normalized time-averaged finger force:

9 $$\overline{s}_{k} = \frac{{\overline{{f_{k} (t)}} }}{{\overline{{f_{1} (t)}} + \overline{{f_{2} (t)}} + \overline{{f_{3} (t)}} + \overline{{f_{4} (t)}} }}$$

where $\overline{{f_{k} (t)}}$ is the time-averaged force at the *k*th finger. As previously mentioned, there were 18 subjects in the experiment and 12 trials for each condition, which results in 216 combinations of sharing ratios. The histogram of sharing ratios results in a skewed distribution. To generalize the observed trends outside the empirical data, we represent the data using a gamma distribution, which has been suggested to adequately describe parameter spaces in human movement science^38^. We perform a maximum likelihood estimation (MLE) analysis^39^ to find the best gamma distribution fitting these combinations of sharing ratios. Table 1 shows the two parameters of the gamma distribution (i.e., shape and scale) for the combinations of the sharing ratios. Finally, in simulating each trial, a set of sharing ratios is randomly chosen from the distributions such that the sum of the sharing ratios of all fingers adds up to one. Random selection of sharing ratios from the distributions allows us to account for differences in performance between subjects and trials. Since the sharing ratio calculated from the experiment did not change between the two tactile feedback conditions, the same ratios were used in the simulation of both conditions. In addition, a zero-mean Gaussian noise (*n*_*s,k*_) is added to the sharing ratio to account for small-amplitude random fluctuations empirically observed, i.e., $s_{k} = \overline{s}_{k} + n_{s,k}$.

**Table 1:** Gamma distribution parameters for fingers’ sharing ratio, variance, and controller design specifications. Shape and scale parameters of gamma distribution for sharing ratio of individual fingers’, variances of individual and total finger force are provided. Similarly, these parameters are given for the controller design specifications, i.e., SSE, %OS, and rise time based on performance specification observed from empirical data.

Variable	Shape parameter	Scale parameter
Sharing ratio for the index finger	15.46	0.02
Sharing ratio for the middle finger	33.96	0.01
Sharing ratio for the ring finger	30.90	0.01
Sharing ratio for the little finger	4.53	0.03
Index force variance	0.44	0.16
Middle force variance	0.47	0.17
Ring force variance	0.42	0.18
Little force variance	0.25	0.33
Total force variance	1.05	0.10
SSE	5.11	0.06
%OS	0.87	5.97
Rise time	2.14	0.19

For each participant, condition and finger pair, Fourier transforms were averaged across trials and scaled to compute the CSD and PSDs, Eq. (10) was used to compute complex coherence, which was then averaged across participants. One spectral Hanning window with a 5 s duration per trial was considered. Lastly, magnitude-squared coherence and phase were computed as the squared absolute value and argument of mean complex coherence (Fig. 4), respectively.

10 $$Complex\,Coherence = \frac{{CSD_{x,y} }}{{\sqrt {PSD_{x} PSD_{y} } }}$$

As Fig. 4 demonstrates, the behavior of signals are different at different frequencies: at low frequencies (below 1 Hz), the signals are anti-phase, which suggests that at low frequencies, every two pairs of signals covary negatively. Although coherence is relatively low (about 0.1) at these frequencies, the power of signals and the CSD between every two signals were high. As we move to higher frequencies (1.6 Hz–2.5 Hz), the coherence becomes higher (around 0.4), power becomes very close to zero, and the phase shift between the two signals at these frequencies is almost zero. These results suggest that the two signals covary positively at higher frequencies, while negatively correlated with each other at lower frequencies. The negative covariation between the two signals relates to the error compensation feature of synergy between fingers. Our result suggests that this feature of synergies exists at low frequencies.

In order to implement these results in the model, we add colored noise to the sharing function and make it time-varying within a trial. A few studies^33,34^ have shown that CNS uses the inherent noise for the coordination of its effectors. They declared that the noise behaves as pink noise, whose power spectral density follows the power law (1/f). The sharing function described above is accordingly modified to incorporate the inherent pink noise characteristics.

To represent the self and lateral inhibition, which ensures the error compensation of motor synergies according to CBC hypothesis^24^, tunable back-coupling loops involving short-latency feedback are added to the model. The back-coupling loops involve gain (*g*_*ij*_*.*

($1 \le i,j \le 4$) for the four fingers) and delay ($\tau_{CBC}$). The initial parameter values for the gain and delay are chosen based on previously reported values^24^, and these parameters are further tuned within the range of physiologically standard values to demonstrate that the mathematical model could be used to robustly reproduce the experimental data. The final values used for the with tactile feedback condition are *g*_*ij*_ = 0.18 ($1 \le i,j \le 4$) and $\tau_{CBC} = 10$ ms, which are close to the values reported in the literature^24^. For the without tactile feedback condition, we hypothesize that the gain and delay are associated with the reduced synergy after removal of tactile feedback. Accordingly, we reduce the gain to *g*_*ij*_ = 0.12 and increase the delay to $\tau_{CBC} = 100$ ms.

The enslaving function is adopted from a previous study^22^. The sensory noises are assumed to be zero-mean, and the variance parameters are found based on the experimental data. The individual finger variances and total force variance for each trial are calculated from the experiments, and gamma distributions for each of these variances are estimated using MLE (Table 1). For each trial, variances for individual fingers are randomly taken from the distributions. The tactile sensory feedback delay ($\tau_{T}$) is set to 40 ms based on nominal values reported in the literature^10^. In accordance with the results reported in previous behavioral science studies (which reported that minimum sensory transduction delay required for a visual or proprioceptive signal to influence an ongoing movement is about 80–100 ms^9^), the visual sensory feedback delay ($\tau_{V}$) is set to 100 ms. Noting that CNS responds to sensory signals with delay when tactile feedback is deprived^8–10^, the $\tau_{T}$ is set to 100 ms to simulate the effects of anesthesia in the without tactile feedback condition^8,40^. The gain and parameters associated with the finger dynamics^26^ ($\gamma_{k}$, $\zeta_{k}$, and $\omega_{n,k}$, *k* = 1,2, 3, 4) are selected from a range of standard values, and slight deviations from the selected values do not break the model’s ability to predict the data.

The last key to the model is the design of a proper controller that can simulate the command that CNS would send to individual fingers to produce forces under different conditions. Therefore, based on the defined parameters, we design a PD-Lag controller for our model. The controller is designed based on performance specifications calculated from experimental data such as steady-state error (SSE) and some transient response specifications such as percentage of overshoot (%OS), rise time, and settling time. We calculate the SSE, %OS, and rise time for each trial and subject for with tactile feedback condition and estimate the gamma distribution again using MLE (Table 1). Then to simulate each trial, we randomly choose a value for SSE, %OS, and rise time from the distributions to account for the possible differences between subjects and trials. The settling time is set to 5 s for all trials, as subjects reached and remained within the variability of one standard deviation after 5 s.

### Closed-loop response of finger force model

In order to find an analytical form for the transfer function of our model, we considered a block diagram of the form of Fig. 2.

The input reference is taken as a constant reference force in the Laplace domain as follows:

11 $$r(s) = \frac{{F_{0} }}{s}$$

where *s* is the Laplace transform variable, and *F*_*0*_ is the desired force, which is set to 20 N, i.e., target force.

The transfer functions for the different blocks of Fig. 2 are given by Eq. (12)

12 $$\left\{ {\begin{array}{*{20}l} {[S]_{j} = \hat{s}_{j} = \left\{ {\begin{array}{*{20}l} {s_{j} ,} \hfill & {{\text{if}}\quad j \in \{ 1,2,3\} } \hfill \\ {{\mathbf{1}} - \sum\limits_{{k = 1}}^{3} {s_{k} ,} } \hfill & {{\text{if}}\quad j = 4} \hfill \\ \end{array} } \right.} \hfill \\ {C = \gamma _{c} e^{{ - \tau _{{CBC}} s}} {\mathbf{11}}^{T} } \hfill \\ {[E]_{{ij}} = e_{{ij}} } \hfill \\ {[G]_{{ii}} = \frac{{\gamma _{i} \omega _{{n_{i} }}^{2} }}{{s^{2} + 2\xi _{i} \omega _{{n_{i} }} s + \omega _{{n_{i} }}^{2} }}} \hfill \\ {[H_{T} ]_{{ii}} = \gamma _{{T_{i} }} e^{{ - \tau _{T} s}} } \hfill \\ {H_{V} = \gamma _{\upsilon } e^{{ - \tau _{V} s}} } \hfill \\ \end{array} } \right.$$

where **1** is a four-dimensional vector of ones, and all remaining parameter values above are known. The blocks *K*, *S*, *C*, *E* are representative of the central nervous system. The controller is chosen as a PD-Lag of the form

13 $$K = (K_{p} + sK_{d} )\frac{{(s + z_{c} )}}{{(s + p_{c} )}}$$

where the parameters *K*_*p*_ > 0, *K*_*d*_ > 0, 0 <|*p*_*c*_| <|*z*_*c*_*|* are designed based on provided performance specifications such as SSE, %OS, settling time and rise time, as previously mentioned.

The self and lateral inhibition (*L*) is represented by an inner feedback loop (i.e., the back-coupling loop), and can be reduced to

14 $$L = (I + C)^{ - 1} = (I + \gamma_{c} e^{{ - \tau_{CBC} s}} {\mathbf{11}}^{T} )^{ - 1} = I - \frac{{\gamma_{c} e^{{ - \tau_{CBC} s}} }}{{{\mathbf{1}} + \gamma_{c} e^{{ - \tau_{CBC} s}} }}{\mathbf{11}}^{T}$$

by virtue of the Sherwood-Morrison inversion lemma^41^.

By utilizing the transfer functions of the individual plants described above in the model, we derive the closed-loop response of the finger force system. This response takes the form of Eq. (15), where we can see the effect of each input source on the output (*Y(s)*).

The output *Y*(*s*) is the combined finger forces generated by our model.

15 $$Y(s) = \frac{{{\mathbf{1}}^{T} G_{1} }}{{G_{3} }}n_{s} + \frac{{{\mathbf{1}}^{T} G_{1} G_{2} }}{{G_{3} }}r - \frac{{{\mathbf{1}}^{T} G_{1} G_{2} \mathbf{1}^{T} H_{T} }}{{G_{3} }}n_{T} - \frac{{{\mathbf{1}}^{T} G_{1} G_{2} H_{v} }}{{G_{3} }}n_{v}$$

where *G*_*1*_, *G*_*2*_, and *G*_*3*_ are as follows

16 $$\begin{aligned} & G_{1} = LEG \\ & G_{2} = KS \\ & G_{3} = {\mathbf{1}} + {\mathbf{1}}^{T} (H_{T} + H_{v} I_{4 \times 4} )G_{1} G_{2} KS \\ \end{aligned}$$

Finally, given the closed-loop transfer function (Eq. (15)) and assigned parameters for different sections, we design a PD-Lag controller (Eq. (13)) to emulate the control role of CNS.

### Controller design

We wish to determine the values of *K*_*p*_ and *K*_*d*_ in Eq. (13) that will satisfy the provided transient response specification. The closed-loop poles of the system are given by the values of *s* that satisfy the characteristic equation.

17 $$1 + K(s)G_{fb} (s) = 0$$

In order to simplify the design process, we perform a model order reduction and approximate the feedback-path transfer function by a second-order system of the following form

18 $$G_{fb} (s) \approx \frac{{b_{0} }}{{s^{2} + a_{1} s + a_{0} }}$$

**Figure 6:**
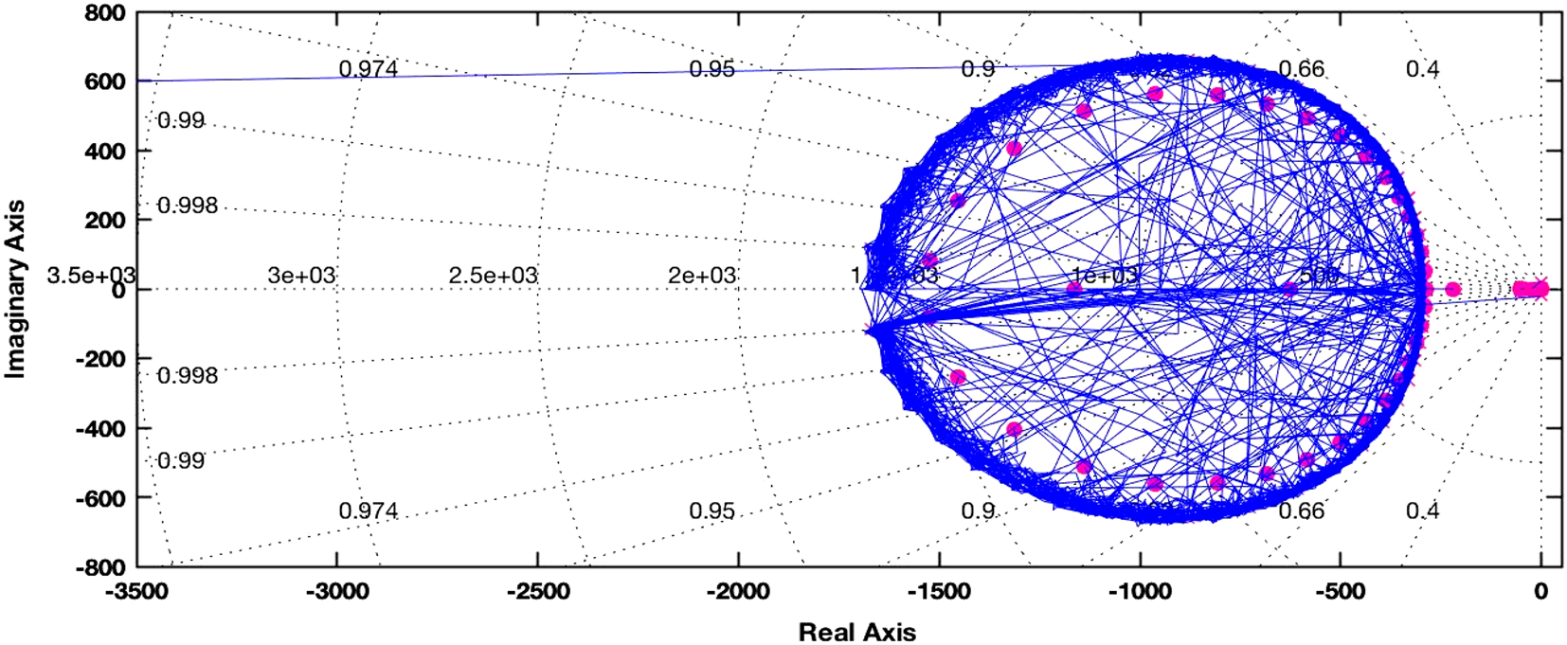
Root locus of the feedback system for a representative subject in one trial. The root locus is a graphical representation in the complex *s*-plane of the possible locations of the poles of the closed-loop system, which dictate the behavior of the system. The poles near the origin are sufficiently separated from the remaining poles that they dominate the performance of the system.

Upon performing a bilinear transform of signal delays, the actual feedback system has 77 states. The root locus for a representative subject in one trial is presented in Fig. 6. It can be seen that the two poles close to the imaginary axis dominate the response of the system. These poles were very close to the poles of the second-order system chosen for modeling the finger dynamics. Hence, the estimation of the behavior of the system through model order reduction becomes feasible. In particular, we choose *a*_*0*_*, a*_1_*, b*_*0*_ according to the finger dynamics in Eq. (4)*.*

The lag compensator pole *p*_*c*_ for each trial is randomly taken from the gamma distribution found for the rise time of empirical responses (Table 1). Finally, the lag compensator zero (*z*_*c*_) is calculated based on specified SSE (*e*_*ss*_), and selected pole (*p*_*c*_) as Eq. (19) obtained from the application of the final value theorem^42^.

19 $$z_{c} = \frac{{(1 - \frac{{e_{ss} }}{{F_{0} }})}}{{K_{p} (\sum\limits_{i = 1}^{4} {\sum\limits_{j = 1}^{4} {\gamma_{i} e_{ij} (\hat{s}_{j} - \frac{{\gamma_{c} }}{{1 + 4\gamma_{c} }})} } - (1 - \frac{{e_{ss} }}{{F_{0} }})\sum\limits_{i = 1}^{4} {\sum\limits_{j = 1}^{4} {\gamma_{i} e_{ij} (\hat{s}_{j} - \frac{{\gamma_{c} }}{{1 + 4\gamma_{c} }})(\gamma_{\upsilon } + \gamma_{{T_{i} }} ))} } }}p_{c}$$

### Data analysis

In order to validate our simulation results, the data collected from experiments and simulations were compared according to the HVD approach^17^. Based on this approach, the task performance is quantified in terms of the overall mean squared error (OMSE) and decomposed to within-trial (called “online” $\overline{{Var(\tilde{y})}}$) and trial-to-trial (called “offline” $Var(\varepsilon )$) variances of the resultant finger force, and systematic error (*r-m*)^*2*^ as:

20 $$OMSE = \frac{1}{N}\sum\limits_{i = 1}^{N} {\frac{1}{\tau }[r(t) - y_{i} (t)]^{2} } = \overline{{Var(\tilde{y})}} + Var(\varepsilon ) + (r - m)^{2}$$

The variances for the resultant finger force were further decomposed into the individual finger level as follows:

21 $$\overline{{Var(\tilde{y})}} = \overline{{Var\left( {\sum\limits_{k = 1}^{4} {\tilde{f}_{k} } } \right)}} = \overline{{\sum\limits_{k = 1}^{4} {Var(\tilde{f}_{k} )} }} + \overline{{\sum\limits_{j \ne k}^{4} {Cov(\tilde{f}_{j} ,\tilde{f}_{k} )} }}$$

and

22 $$Var(\varepsilon ) = Var\left( {\sum\limits_{k = 1}^{4} {\varepsilon_{k} } } \right) = \sum\limits_{k = 1}^{4} {Var(\varepsilon_{k} )} + \sum\limits_{j \ne k}^{4} {Cov(\varepsilon_{j} ,\varepsilon_{k} )}$$

where $\tilde{f}_{k}$ and $\varepsilon_{k}$ are demeaned force and force averaged over time, respectively, corresponding to the finger k. In Eqs. (21) and (22), the covariance terms represent the within-trial (Eq. (21)) and trial-to-trial (Eq. (22)) interactions of multiple fingers during force production, thus they are used as a measure of multi-finger synergy associated with the force production task. For each subject, HVD is evaluated for both with and without tactile feedback conditions. In each trial, the 5–11 s window of data is extracted for the analysis. The influence of tactile sensory feedback on multi-finger synergy is examined by analyzing the OMSE and its components for with and without tactile feedback conditions.

### Statistical analysis

In order to validate our model with the experiment using the HVD method, we used t-test to assess the differences between the two conditions for the model and experiment. The level of statistical significance was set to *p* = 0.05.

## Supplementary Information

Supplementary Information.

## Data Availability

The data set and model analyzed and generated during the current study are available from the corresponding author upon reasonable request. All the data and design parameters needed for evaluation of the conclusions are provided in the paper.
