# Supplementary material for: Unveiling the neuromechanical mechanisms underlying the synergistic interactions in human sensorimotor system
Source: Sci Rep. 2021 Jan 8;11:203. doi: 10.1038/s41598-020-80420-z (PMC7794444; doi:10.1038/s41598-020-80420-z)
Supplement: Supplementary file 1 — Supplementary Information. [file 41598_2020_80420_MOESM1_ESM.docx]

Title

Unveiling the Neuromechanical Mechanisms Underlying the Synergistic Interactions in Human Sensorimotor System

**Authors**

S. Honarvar^1, †^, C. Kim^2, †^, Y. Diaz-Mercado^1, *^, K. Koh^3^, H. J. Kwon^4^, T. Kiemel^4, 5^, M. Caminita^4^, J. O. Hahn^1^, J. K. Shim^4, 5, 6, *^

**Affiliations**

^1^Department of Mechanical Engineering, University of Maryland, College Park, MD 20742, USA

^2^School of Mechanical Engineering, Chonnam National University, Gwangju, 61186, South Korea

^3^School of Medicine, University of Maryland, Baltimore, MD 21201, USA

^4^Department of Kinesiology, University of Maryland, College Park, MD 20742, USA

^5^Program in Neuroscience & Cognitive Science, University of Maryland, College Park, MD 20742, USA

^6^Department of Mechanical Engineering, Kyung Hee University, Yongin-Si, Gyeonggi-do, South Korea

^†^ These authors contributed equally to this work

^*^ Co-corresponding author. Email: [yancy@umd.edu](mailto:yancy@umd.edu), [jkshim@umd.edu](mailto:jkshim@umd.edu)

**Supplementary Information**

In this section, we present the HVD results of a number of simulations that were performed to examine the effects from variations in the model and further support the conclusions in the paper.

**Fig. S1. Simulation results after removing the tactile loop from the model.** Unlike the experimental results, removal of tactile feedback results in decrease in OMSE, systematic error and offline variance after removal of tactile feedback.

**Fig. S2. Simulation results after increasing the delay and reducing the tactile gain in Bayesian sensory integration.** We see that although OMSE, $Var(\varepsilon)$, and (r-m)^2^ remained unchanged in both conditions, the model cannot reproduce the trend in online synergy.

**Fig. S3. Simulation results after removing CBC.** We can see the results of simulation for both conditions are not different. And removing CBC not only causes the covariance to become positive for both cases but it cannot explain the deterioration of synergy due to sensory deprivation.

**Fig. S4. Simulation results with fixed sharing ratio without component for creating drift (i.e., no pink noise and lag compensator).** We can see that the model performs poorly almost everywhere. Specially, the online and offline synergy terms became positive and the error terms are very large, which indicates why counter-directional drift is important.
